# Screening differential circular RNA expression profiles reveals the regulatory role of circMARS in anti‐tuberculosis drug‐induced liver injury

**DOI:** 10.1111/jcmm.17157

**Published:** 2022-01-14

**Authors:** Biao Li, Qi Ren, Yuhong Li, Shenqian Tian, Yingzhi Chong, Shufeng Sun, Fumin Feng

**Affiliations:** ^1^ School of Public Health North China University of Science and Technology Tangshan China; ^2^ Xiaoshan District Center for Disease Control and Prevention Hangzhou China; ^3^ College of Nursing and Rehabilitation North China University of Science and Technology Tangshan China; ^4^ School of Life Science North China University of Science and Technology Tangshan China

**Keywords:** biomarker, circular RNA, drug‐induced liver injury, function axis, tuberculosis

## Abstract

Tuberculosis (TB) treatment is plagued by liver damage, which often leads to treatment interruptions. Circular RNAs (circRNAs) are a special class of non‐coding RNAs abundant in body fluids with important biological functions. However, the role of circRNA in anti‐tuberculosis drug‐induced liver injury (ADLI) is unclear. We explored ADLI‐specific circRNAs in TB patients using circRNA microarrays and verified circMARS in a cohort of 300 individuals. In addition to the value assessment of circMARS in patients using a receiver operating characteristic (ROC) curve, cell experiments were also performed under the guidance of bioinformatics analyses. In particular, we found that circMARS acts as a miRNA sponge by binding to miRNAs. Compared with the blank group, the expressions of circMARS, KMT2C gene, and EGFR protein in the ADLI group were increased, while miR‐6808‐5p, miR‐6874‐3p, and miR‐3157‐5p were decreased. Furthermore, when si‐circMARS was used in the ADLI groups, circMARS demotion manifested the opposite results. Subsequently, a self‐controlled cohort of 35 participants was used to verify the circMARS–miR‐6808‐5p/‐6874‐3p/‐3157‐5p–KMT2C–EGFR function axis. Therefore, circMARS may participate in the compensatory repair mechanism of ADLI through the function axis, and may be a potential biomarker for ADLI diagnosis in TB patients.

## 1 INTRODUCTION

Tuberculosis (TB) is a chronic infectious disease caused by *Mycobacterium tuberculosis*. It is a preventable and treatable disease mainly transmitted from person to person through the air. Globally, China has a high burden of TB and the third‐highest incidence behind India and Indonesia. According to the global tuberculosis report (2019) by the World Health Organization (WHO), there were about 866,000 TB cases and 40,000 TB deaths in China.^1^ One of the health‐related goals of sustainable development is to curb the TB epidemic by 2030. The World Health Assembly passed the resolution with ambitious targets, but it is not an easy promise. Standardized chemotherapy is a crucial means to achieve this goal. However, the adverse effects of chemotherapeutic drugs such as anti‐tuberculosis drug‐induced liver injury (ADLI) hamper the achievement of the treatment goals. Thus, it is of great significance to study the pathogenesis of ADLI. Previous studies have reported that methylation, acetylation, and non‐coding RNAs in epigenesis are closely related to ADLI. However, further studies on the role of circRNAs in ADLI are needed.^2^, ^3^, ^4^, ^5^

Genetic alterations are the key to revealing the mechanism of ADLI. Circular RNA (circRNA) is a class of novel RNAs commonly transcribed from genome. CircRNA differs from linear RNA, as it has a covalently closed loop structure without the 5′‐cap and 3′poly‐A tail.^6^, ^7^ CircRNA possesses characteristics of universality, stability, specificity, and conservatism; thus, many circRNAs have been found and reported as excellent biomarkers in the past few years.^8^, ^9^ CircRNA has been reported to have good potential as a biomarker for malignancies, cardiovascular diseases and active TB.^10^, ^11^, ^12^ The use of biomarkers has become one of the important methods for the diagnosis and prognosis of various diseases. Abnormal expression of circRNA may participate in the pathogenesis of various human diseases. However, the pathogenesis role of circRNAs has not been investigated in ADLI. Moreover, the biogenesis and potential functions of circRNAs remain unknown. Thus, studies on the diagnostic markers and the effectiveness of complete standardized treatment in TB are important.

Recent studies have demonstrated that circRNAs have miRNA sponge effects and regulate gene expression by microRNA response elements.^13^, ^14^ This study aims to investigate the circRNAs abnormally expressed in TB and to explore their function in ADLI. A two‐phase screening/validation project was performed in patients on anti‐TB treatment. In the screening phase, the circRNA expression profiles in the serum were assessed using a human circRNA microarray, which was designed to simultaneously detect thousands of circRNAs, in 16 patients with ADLI and 16 non‐ADLI patients matched for age, gender, and treatment. In the validation phase, the circMARS in the serum was validated by quantitative real‐time polymerase chain reaction (qRT‐PCR) in a cohort of 300 patients. The involvement of circRNAs in liver damage related pathways via interactions with miRNAs was then investigated by multiple bioinformatics approaches. The findings of the bioinformatics analyses were verified in cytology experiments and a self‐controlled cohort of 35 patients.

## 2 MATERIALS AND METHODS

### 2.1 Subjects design

This study enrolled patients diagnosed with TB at Tangshan Tuberculosis Hospital (China) from July 2015 to July 2018. All patients were put on the same TB therapeutic regimen (daily 2S(E)HRZ/4HR: S, streptomycin; E, ethambutol; H, isoniazid, R, rifampicin; Z, pyrazinamide; dose increased for 2 months and then consolidated for 4 months). ADLI was defined according to the Danan Criteria promulgated in 1990. The inclusion criteria for the ADLI group were liver injury following 6 months of anti‐TB drug therapy, while for the non‐ADLI group, it was the absence of liver injury following 6 months of anti‐TB drug therapy. The patient exclusion criteria were patients diagnosis of other liver diseases, use of other drugs that cause abnormal liver function, and abnormalities in liver structure or function before anti‐TB treatment. This study was approved by the Ethics Committee of North China University of Science and Technology. All of the selected patients provided informed consent, and the study protocol conformed to the ethical guidelines of the Declaration of Helsinki (1975).

**FIGURE 1:**
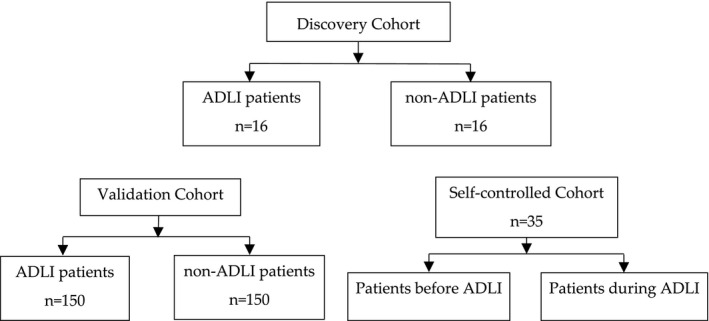
Study flow chart. First, the difference of circRNA expression profiles between anti‐tuberculosis drug‐induced liver injury (ADLI) and non‐ADLI patients were explored. Then, a differentially expressed circRNA was selected for verification in a cohort of 300 patients. Finally, the function of this circRNA was verified in a self‐controlled cohort of 35 patients based on the results of the experimental study

This study was divided into three phases. Sixteen ADLI patients were selected and matched with 16 non‐ADLI patients for age and gender. Total RNAs were then isolated from their peripheral blood for microarray expression profiling. Furthermore, the expression profiles of circRNAs were also detected in the ADLI group. Then, the circRNAs were verified in the second phase, and its diagnostic value was investigated. The validation cohort consisted of 150 ADLI patients and 150 non‐ADLI patients. Finally, cytology experiments were conducted, and a self‐controlled cohort of 35 participants before and during ADLI was used to determine the function of circRNA. The detailed flowchart of this study is shown in Figure 1.

### 2.2 RNA isolation and circRNA microarray expression profiling

Fresh peripheral venous blood samples were centrifuged, and the serum was collected from the upper layer. Subsequently, total RNA was extracted from the serum and the cell experiments using TRIpure reagent (BioTeke, Beijing, China), according to the manufacturer's instructions. The circRNAs in the discovery cohort were profiled using the CapitalBio Technology CircRNA Human Gene Expression Microarray v2.0 (CapitalBio Technology, Beijing, China). The circRNA array data were analyzed using the GeneSpring software V13.0 (Agilent). The data were Log2 transformed and median centred by circRNAs using CLUSTER 3.0 software to determine the differentially expressed circRNAs. The differential expression of the circRNAs between both groups was evaluated by assessing the change in the two‐fold change (FC > 2) and a *T*‐test *p* value of 0.05. The *p* value was corrected by Benjamini‐Hochberg method.

### 2.3 Expression quantification and identification of circRNA

qRT‐PCR was performed using a GoScript Reverse Transcription Mix kit (Promega, Madison, USA) and a TB Green Premix Ex Taq II kit (TaKaRa, Dalian, China). Briefly, the total RNA was reverse transcribed into the cDNA using the PCR Thermal Cycler system (Bio‐Rad, Hercules, USA): incubation at 42°C for 1 h, 95°C for 5 s, and 4°C until the end of the experiment. The conditions of the RNA expression quantification on the ABI Step One Plus system (Applied Biosystems, Carlsbad, USA) were as follows: denaturing initially at 95°C for 30 s, followed by 50 cycles of denaturation at 95°C for 30 s, annealing at 60°C for 30 s. Divergent primers for circMARS and other convergent primers for PCR were designed and synthesized by Sangon Biotech (Shanghai, China) Co., Ltd.. The primer sequences are shown in Table S1. Glyceraldehyde‐3‐phosphate dehydrogenase (GAPDH) was used to normalize the relative expression levels of circRNA and mRNA, and small nuclear U6 was used to normalize the miRNA expression levels. The relative expression levels of the RNA calculation were calculated using the 2^−ΔΔCt^ method. Furthermore, the PCR products were cut using a gel electrophoresis experiment and were sent to SinoGenoMax (Beijing, China) Co., Ltd., for the sanger sequencing.

### 2.4 Inferring circRNAs pathways and network

In order to further explore the biological functions of circMARS with a differential expression in ADLI, miRanda 3.0 and TargetScan 7.0 were used to predict the target miRNA of circMARS. Moreover, Kyoto Encyclopedia of Genes and Genomes (KEGG) pathway analysis and Gene Oncology (GO) enrichment analysis of these miRNAs were performed with mirPath v3.0.

### 2.5 Vitro assays

HL‐7702 human hepatocytes (OBiO, Shanghai, China) were cultured in Roswell Park Memorial Institute (RPMI) 1640 medium with 10% fetal bovine serum (FBS; Gibco, New York, USA) and 1% penicillin/streptomycin (Sigma‐Aldrich, St. Louis, MO, USA) at 37°C in a cell incubator with 5% CO_2_. The cell cultures were divided into six groups: (i) Blank group: only cells; (ii) siRNA‐control group (si‐ctrl): medium containing negative control siRNA; (iii) two drugs group (twd): medium containing 88 µg/mL INH + 176 µg/mL RFP; (iv)three drugs group (trd): medium containing 54 µg/mL INH + 109 µg/mL RFP + 292 µg/mL PZA; (v) si‐circMARS + two drugs group (si + twd): medium containing 50 nmol/L si‐circMARS and the prescribed concentration of two drugs; (vi) si‐circMARS + three drugs group (si + trd): medium containing 50 nmol/L si‐circMARS and the prescribed concentration of three drugs. The survival rate of the cells treated with drugs was measured after 48 h, using the Cell Counting Kit‐8(CCK8). The blank group, twd group, and trd group were selected for microarray screening of the circular RNA expression profiles. The siRNAs of the circMARS and control were transfected with Lipofectamine 2000 (Invitrogen, Carlsbad, USA), following the manufacturer's instructions.

### 2.6 Luciferase reporter assay

GP‐miRGLO‐circMARS‐wt/mut, miRNAs mimics (miR‐mimics) and control (miR‐NC) were constructed by GenePharma (Soochow, China). They were co‐transfected into HL‐7702 cells using Lipofectamine 2000. Luciferase activity was measured at 48 h post‐transfection by Dual‐Luciferase Reporter Assay System (Promega, Madison, WI, United States). The relative luciferase activity was normalized to the control group (miR‐NC).

### 2.7 ALT, AST activity and EGFR protein assays

The ALT and AST in the cell supernatant were detected by the Reitman‐Frankel method kit (Nanjing Jiancheng Bio, Nanjing, China). The epidermal growth factor receptor (EGFR) concentration in serum was measured by enzyme linked immunosorbent assay (Abcam, Cambridge, UK) according to the manufacturer's instructions.

### 2.8 Statistics

Data were analyzed by SPSS 22.0 software (IBM, Chicago, USA). GraphPad Prism 5.0 software (GraphPad Software, San Diego, USA) and Excel 2016 software (Microsoft, Redmond, USA) were used to generate graphs. Student's *t*‐test and one way analysis of variance (ANOVA) test were used as appropriate. The clinical diagnostic value of a given circRNA was verified by ROC curve analysis. The value of *p* < 0.05 (two‐sided) was regarded as statistically significant for all of the statistical calculations.

## 3 RESULTS

### 3.1 Identification of circRNA expression profile in ADLI

**FIGURE 2:**
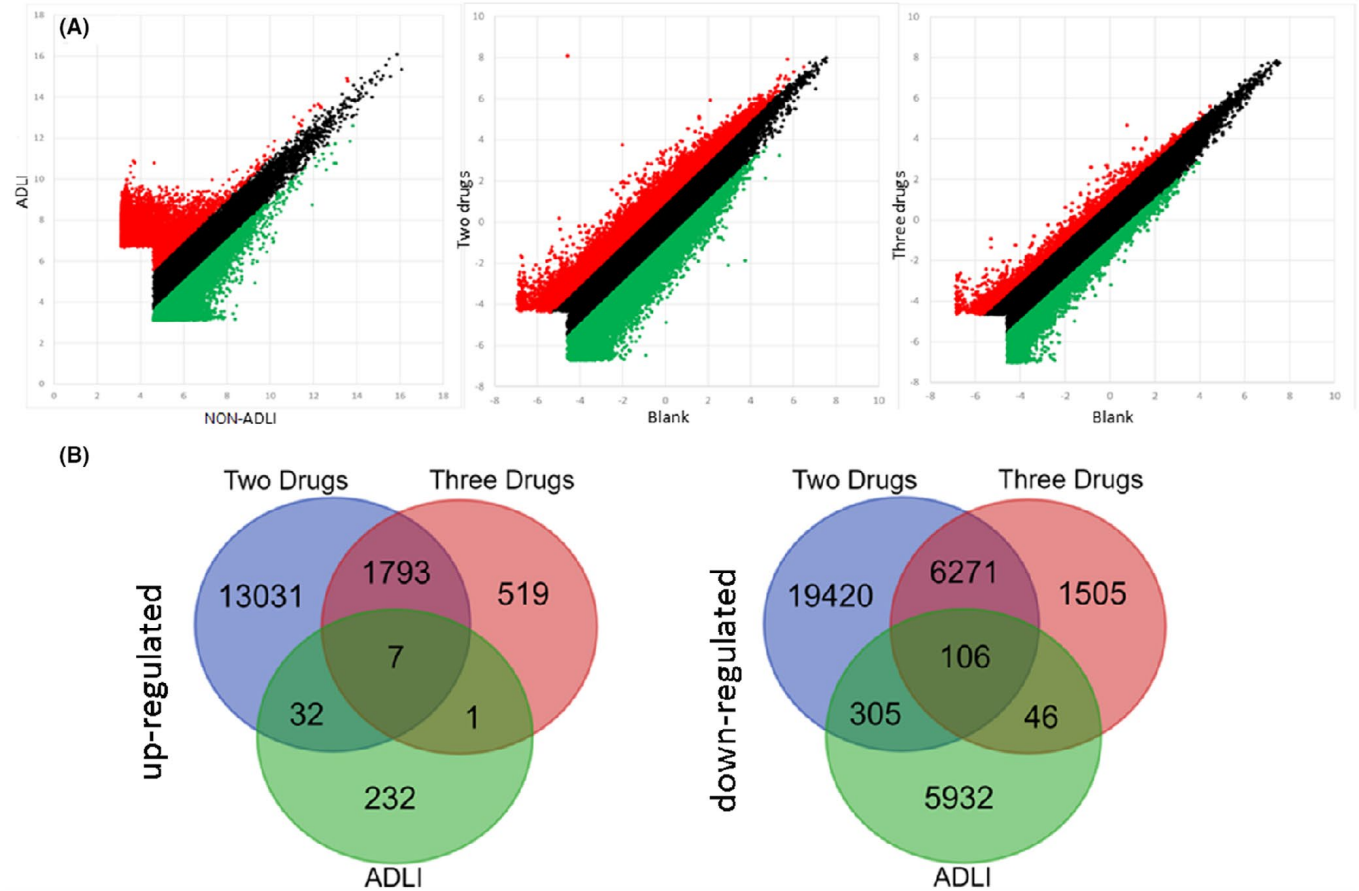
Identification of circRNA expression profiles in ADLI. (A) The scatter plots of circRNAs expression variations in the ADLI patients and in vitro assays. The red and green points in the plot indicate the upregulated and downregulated circRNAs. (B) Venn diagrams of the co‐expression circRNAs in the serum and cells. Two drugs: cells with Isoniazid (INH) + Rifampicin (RFP); three drugs: cells with INH + RFP + Pyrazinamide (PZA)

In the first phase of the study, circRNA expression profile was detected using circRNA microarray in patients’ serum and the three cell experiment groups (blank group: normal cell; two drugs group: cell with Isoniazid (INH) + Rifampicin (RFP); three drugs group: cell with INH + RFP + Pyrazinamide (PZA)). A total of 6661 differentially expressed circRNAs were identified in the patients’ serum, which included 272 that were upregulated and 6389 were downregulated. However, in the cell experiments, there were 8177 differentially expressed circRNAs that included 1800 that were upregulated and 6377 downregulated. The differentially expressed circRNAs are shown in Figure 2a. Patient characteristics on gender, age, and body mass index (BMI) were comparable between the groups (Table S2). All patients were newly diagosed with TB with no previous history of smoking, drinking or other chronic diseases.

The intersection results of the two microarrays showed 113 co‐differentially expressed circRNAs, which included seven that were upregulated and 106 that were downregulated (Figure 2b and Table S3).

### 3.2 Verification of expression levels and the potential value of circMARS

**FIGURE 3:**
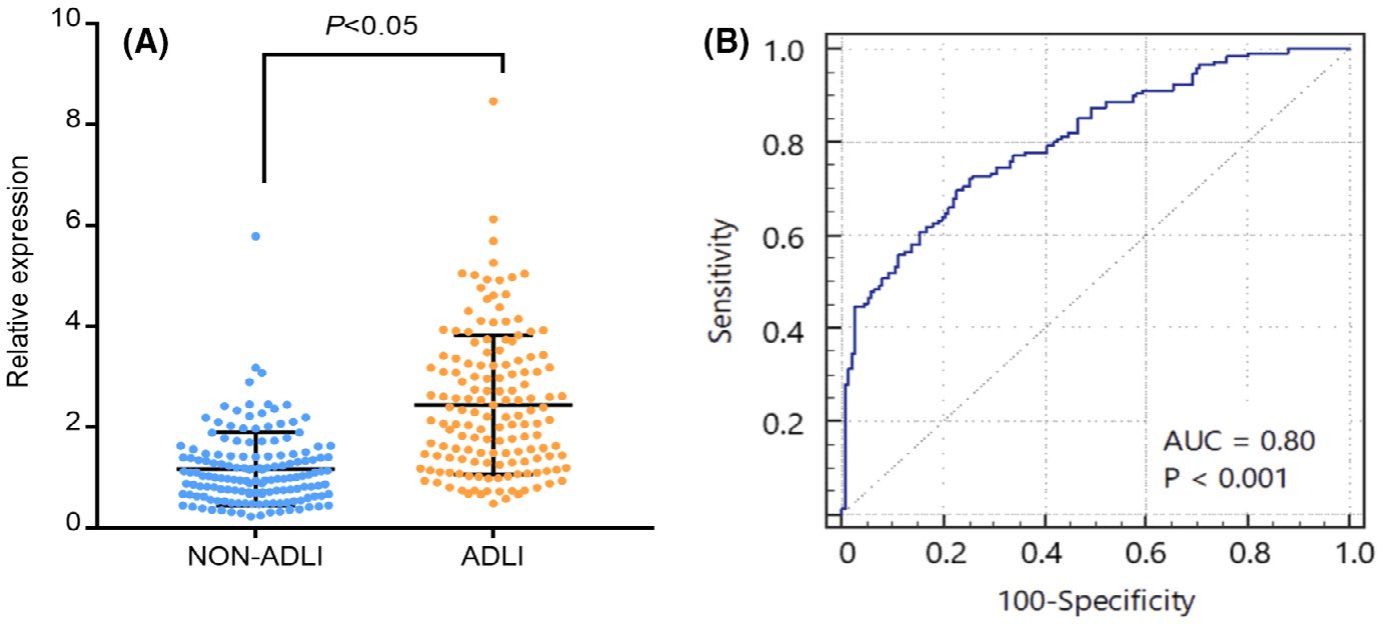
Verification of the expression level and potential diagnostic function of circMARS in 300 tuberculosis (TB) patients. (A) The relative expression level of circMARS in the ADLI and non‐ADLI patients was measured by qRT‐PCR. (B) The receiver operating characteristic (ROC) curve of the ADLI index in distinguishing ADLI patients from non‐ADLI TB patients

Microarrays have the advantages of high flux and efficiency. However, they are prone to false positives and low stability. Considering the abundance level and biological analysis of circRNA, we selected circMARS (circBase ID: hsa_circ_0027252) for qRT‐PCR validation. A total of 300 patients were included in the study, with 150 patients allocated to the ADLI group and 150 patients allocated to the non‐ADLI group. There were no significant differences in clinical characteristics (such as age, gender, BMI, smoking, drinking, and education level) between the two groups, as shown in Table 1. Furthermore, the PCR products show that circMARS was resistant to RNase R and sequenced by the Sanger method. The sequence information was consistent with circMARS reported in the circBase database (Figure S1). The qRT‐PCR results of the serum samples showed that the expression of circMARS in the ADLI group was higher than the non‐ADLI group, and the difference was statistically significant (Figure 3a).

**TABLE 1:** Comparison of basic information of the two groups

Basic information		ADLI (*n* = 150)	NON‐ADLI (*n* = 150)	*χ^2^*/*t*	*P*
Age (year)		46.11 ± 17.33	48.88 ± 19.89	0.182	0.917
BMI (kg/m^2^)		17.37 ± 2.34	17.84 ± 2.23	1.744	0.084
Gender	Male	113	107	0.614	0.433
	Female	37	43		
Smoking	Yes	42	29	3.118	0.077
	No	108	121		
Drinking	Yes	31	24	1.091	0.296
	No	119	126		
Education	Elementary school and below	53	67	3.940	0.139
	Middle school	65	49		
	College and above	32	34		

To further estimate the diagnostic value of circMARS in ADLI, a receiver operating characteristic (ROC) curve was constructed to differentiate the ADLI from the non‐ADLI group. The area under curve (AUC) was 0.80 (95% confidence interval: 0.66–0.87, *p* < 0.05), with a sensitivity and specificity of 0.70 and 0.77, respectively (Figure 3b). These results suggest that circMARS has a good diagnostic value in ADLI and offers a basis for further studying ADLI‐related circRNA.

### 3.3 Inference for biological functions of circMARS in ADLI

CircMARS, 381nt, is derived from the MARS gene (NM_004990) by circularization of the 3~6 exon.^15^, ^16^ CircMARS acts as a miRNA sponge and can bind 98 miRNAs (Table S4). Five of these miRNAs (miR‐3157‐5p, miR‐6808‐5p, miR‐6874‐3p, miR‐4743‐3p, and let‐7e‐5p) have multiple binding sites on circMARS. The Bioinformatics analyses of these miRNAs revealed that miR‐3157‐5p, miR‐6808‐5p, and miR‐6874‐3p were associated with the lysine degradation pathway (hsa00310) and histone methyltransferase activity (GO: 0042800; Figures 4, S2, and S3) through KMT2C (FDR < 0.05). KMT2C regulates the expression of EGFR, which plays an essential role in liver regeneration and repair. Therefore, the circMARS–miR‐6808‐5p/6874‐3p/3157‐5p–KMT2C–EGFR functional axis was selected for further studies.

**FIGURE 4:**
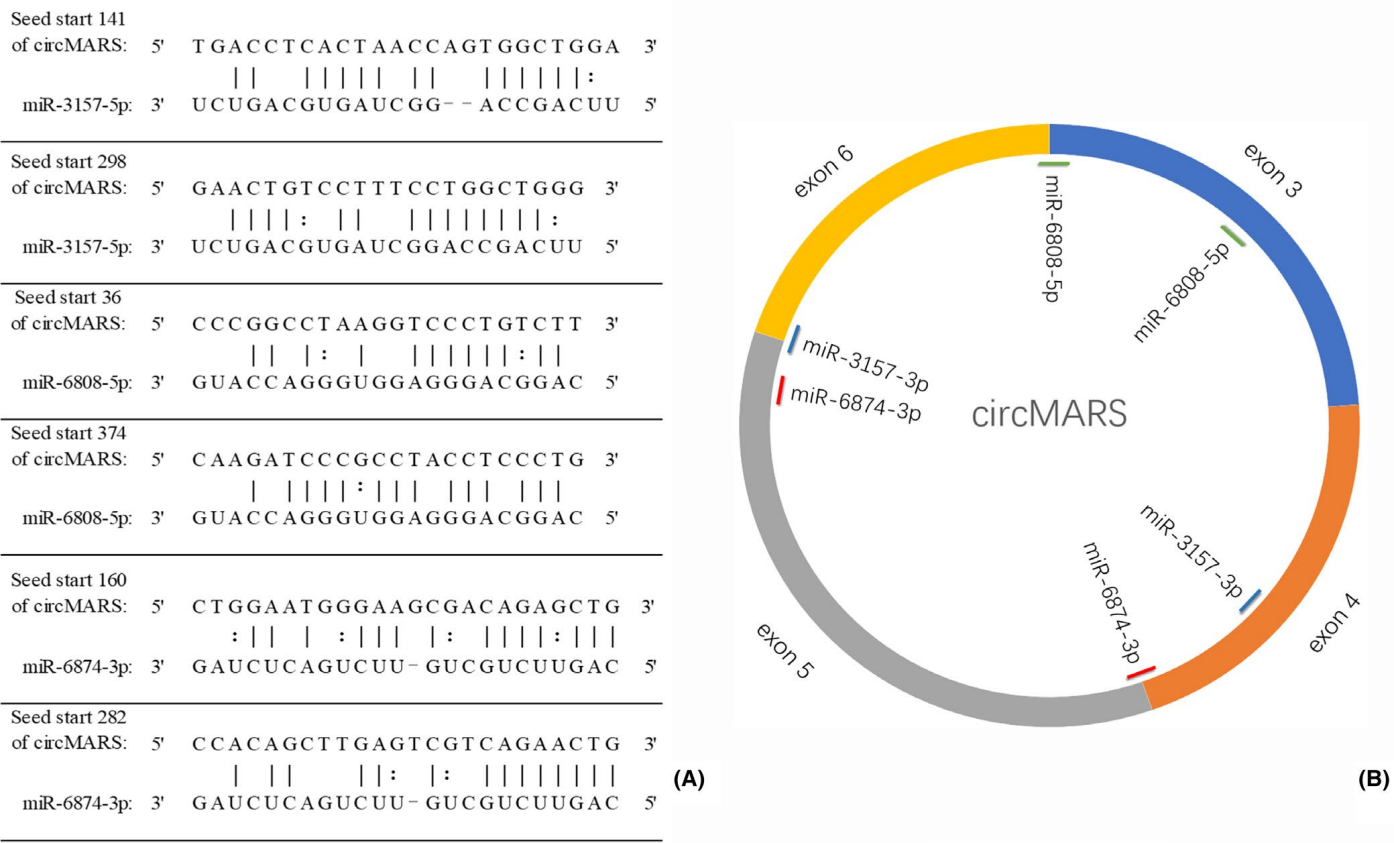
Inference of circMARS biological functions in ADLI. CircMARS has many sponge miRNAs, and some miRNAs have multiple binding sites on it. (A) The sequences of miR‐3157‐5p, miR‐6808‐5p, and miR‐6874‐3p binding to circMARS were predicted. (B) Schematic diagram of three miRNAs targeting circMARS

### 3.4 CircMARS function in cells

**FIGURE 5:**
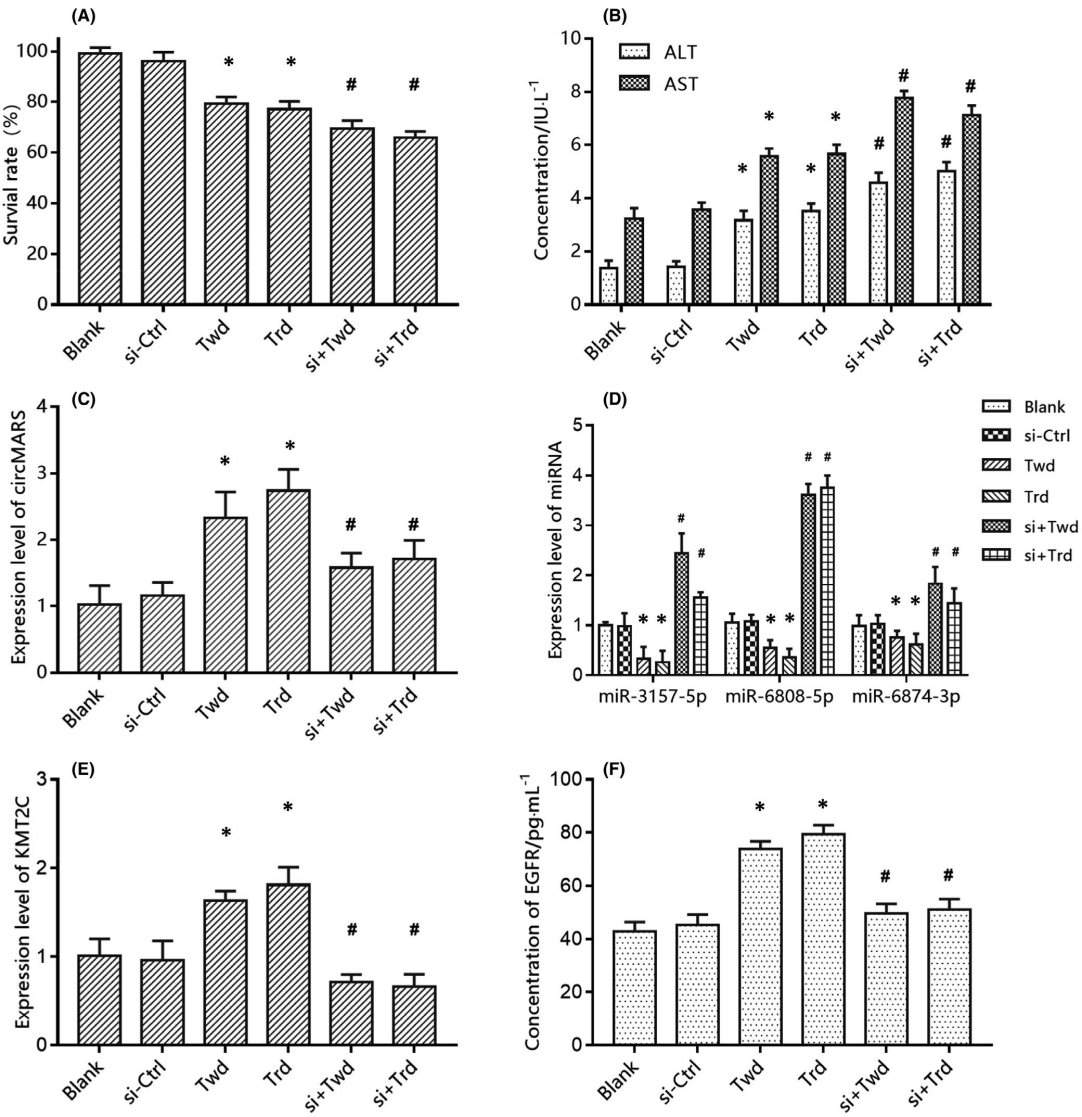
In vitro determination of circMARS function axis. CircMARS plays a regulatory role through the circMARS–miR‐3157‐5p/‐6808‐5p/‐6874‐3p–KMT2C functional axis. (A) Comparison of the survival rate of the cells in different groups. (B) Comparison of the concentration of ALT and AST in different groups. (C–E) Comparison of the expression level of circMARS, miRNAs, and KMT2C in different groups. (F) Comparison of the concentrations of EGFR in different groups. Blank: only cells; Si‐ctrl: the control of siRNA for circMARS; twd: two drugs (INH + RFP); trd: three drugs (INH + RFP + PZA); si + Twd: siRNA of circMARS + twd; si + Trd: siRNA of circMARS + trd. The error bars represent the standard error of the mean. *p < 0.05 versus blank group, #*p* < 0.05 versus their respective drug groups

The dual‐luciferase reporter assay was used to verify the interaction between circMARS and miR‐3157‐5p/miR‐6808‐5p/miR‐6874‐3p. Results revealed that miRNAs mimics markedly reduced the luciferase activity of the circMARS reporter (Figure S4). In addition, the CCK8 assay was used to explore the effects of anti‐TB drugs and circMARS on the survival rate of hepatocytes. As can be seen from Figure 5a, cells in the drugs‐treated groups died in differing degrees compared with the blank group. The survival rates in the twd groups and the trd groups were 79.24% and 77.18%, respectively (*p *< 0.05). Suppression of circMARS by siRNA resulted in a decreased cell survival rate of 69.33% and 65.79%, in the si‐twd group and the si‐trd group, respectively. The difference was statistically significant (*p *< 0.05). Furthermore, ALT and AST levels in the drug‐treated groups were significantly increased than in the blank group (Figure 5b), and hepatotoxicity was observed (*p *< 0.05). However, no changes in the ALT and AST levels were observed in the siRNA transfection negative control siRNA (*p *> 0.05). In contrast, the ALT and AST levels were increased in the circMARS knockdown groups (*p* < 0.05). This finding suggests that a decrease in circMARS leads to a further worsening of ADLI.

The expression level of circMARS in the drug‐treated groups was much higher than in the blank group (*p *< 0.05), which was consistent with the results of the circRNA microarray profile (Figure 5c). SiRNA transfection successfully knocked down circMARS (*p *< 0.05). As expected, the expression of miR‐3157‐5p, miR‐6808‐5p, and miR‐6874‐3p in the drug‐treated groups was decreased compared with the blank group (*p *< 0.05). Meanwhile, the relative expression of miRNAs in the drug‐treated + si‐circMARS groups was significantly higher than the drug‐treated only groups (*p *< 0.05; Figure 5d). Furthermore, as shown in Figure 5e, the expression of the target gene KMT2C increased in the drug‐treated groups and decreased in the drug‐treated +si‐circMARS groups (*p *< 0.05). This finding is consistent with the circMARS expression trend in microarrays, indicating that circMARS plays a regulatory role through the circMARS–miR‐3157‐5p/‐6808‐5p/‐6874‐3p–KMT2C functional axis. EGFR plays an essential role in liver regeneration and repair. As can be seen from Figure 5f, the EGFR levels in the supernatant of the drug‐treated groups were significantly higher than the blank group. However, EGFR was suppressed in the siRNA transfected groups (*p *< 0.05).

### 3.5 Verification of circMARS—miRNAs—KMT2C—EGFR function axis in ADLI patients

**FIGURE 6:**
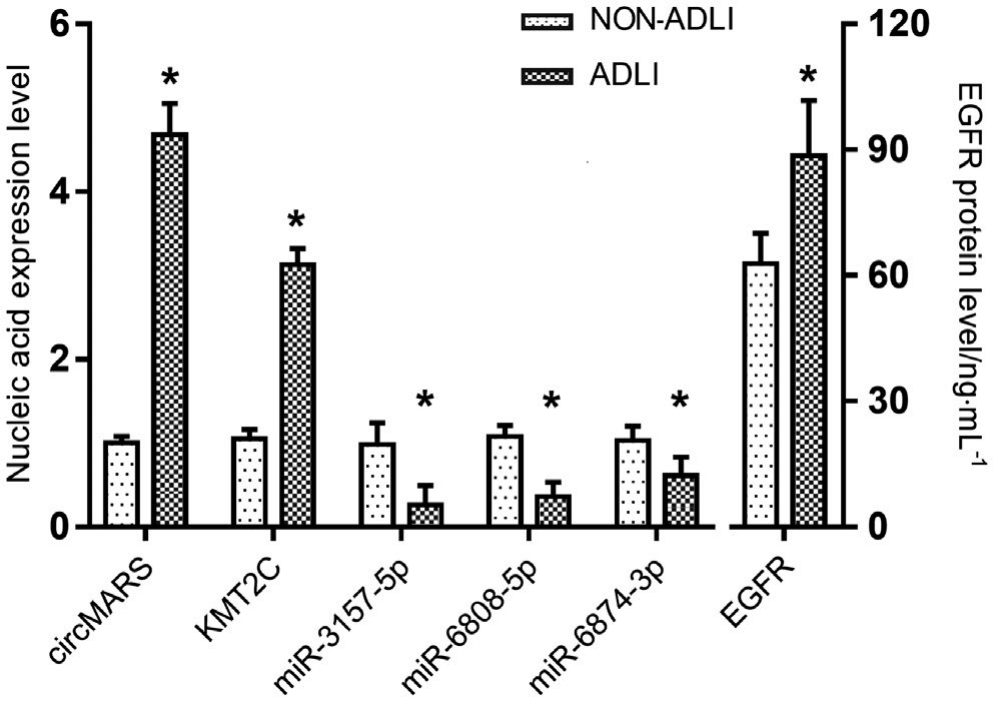
Verification of circMARS function axis in self‐controlled cohort. The serum expression levels of relevant markers before and during ADLI were consistent with that in vitro. The error bars represent the standard error of the mean. *p < 0.05 vs. the non‐ADLI group

This study hypothesized that circMARS–miR‐3157‐5p/‐6808‐5p/‐6874‐3p–KMT2C–EGFR function axis is involved in the liver compensatory repair mechanism in ADLI. A total of 35 patients were selected to act as a self‐controlled cohort to test this hypothesis. All 35 patients were studied before and during ADLI (the basic information of the patients can be found in Table S5). The relative expression of circMARS and KMT2C mRNA in the serum of patients with ADLI was significantly higher than before ADLI. The relative expression of miR‐3157‐5p, miR‐6808‐5p, and miR‐6874‐3p was significantly lower in the patients’ serum during ADLI than before the occurrence of ADLI (Figure 5). EGFR is involved in the occurrence and progression of liver cirrhosis and hepatocellular carcinoma and the regeneration and repair of acute and chronic liver injuries. EGFR protein expression levels in serum before and during ADLI detected by enzyme‐linked immunosorbent assay (ELISA) were (62.81 ± 7.19) ng/mL and (88.53 ± 8.20) ng/mL, respectively, with statistically significant differences (*t* = 3.15, *p* < 0.05; Figure 6). The results of qRT‐PCR and ELISA were consistent with the microarray and cytology experiments results. Thus, circMARS–miRNAs–KMT2C–EGFR function axis may be involved in the compensatory repair mechanism in ADLI.

## 4 DISCUSSION

Drug‐induced liver injury is a significant obstacle in the treatment of TB. Previous studies have investigated non‐specific factors causing ADLI, such as interleukin (IL) and tumour necrosis factor (TNF).^2^, ^17^, ^18^, ^19^ However, the underlying mechanism of drug‐induced liver injury with anti‐TB drugs needs further exploration to develop novel therapeutic targets. The present study documented the circRNA expression profiles of the serum in anti‐TB patients with liver injury for the first time. We aimed to identify a novel biomarker and therapeutic target for ADLI. In addition, we aimed to explore the potential function of circMARS in ADLI. We performed a microarray analysis of dysregulated circRNAs by comparing the circRNA profile in ADLI patients with the controls. A total of 113 circRNAs were differentially expressed, which included seven that were upregulated and 106 that were downregulated. These observations may be helpful in future pathophysiological studies of ADLI and the determination of the role of circRNAs as a non‐invasive biomarker for the diagnosis and treatment of ADLI.

Clinical qualification of biomarkers requires good accuracy, reproducibility, and applicability. The closed loop structure of circRNA makes it highly conserved and remarkably stable, and thus, it is not degraded by exonucleases.^20^, ^21^ It is significantly superior to linear RNAs as a biomarker in diseases. Therefore, there are great advantages in exploring the use of appropriate circRNA biomarkers for liquid biopsy. Moreover, studies have reported on the potential role of circRNA as biomarkers in human diseases. Li et al.^22^ reported abundant exo‐circRNAs in exosomes, representing a new class of stable RNA. Wu et al.^23^ screened the circRNA expression profile in plasma by microarray analysis. They found that circ_0009582, circ_0037120, and circ_0140117 might serve as potential biomarkers for predicting the occurrence of hepatocellular carcinoma (HCC) in patients with HBV infection. Furthermore, He et al.^24^ reported that exosomal hsa_circ_0087862 and hsa_circ_0012077 in cerebrospinal fluid (CSF) are potential diagnostic biomarkers of immune‐mediated demyelinating disease. Bahn et al.^25^ also identified >400 circRNAs in human cell‐free saliva. CircRNA is abundant in body fluids, including blood, cerebrospinal fluid, and saliva. In this study, circRNAs differentially expressed in ADLI were screened by microarray, and circMARS was successfully verified in 300 patients treated with anti‐TB drugs. Results showed that the expression level of circMARS was significantly increased in the serum of ADLI patients. In addition, the reported AUC of 0.80 adds value to the current diagnosis of ADLI. The high accuracy of the circMARS expression profile offers the potential of complementing the currently used indicators for clinical diagnosis and monitoring of ADLI.

Accumulating evidence shows that circRNA has been implicated in various biological processes through the regulation of mRNA expression.^26^, ^27^, ^28^ CircRNA adsorbs miRNAs, and thus, blocks their inhibitory effect on the target mRNAs. CircRNA‐related functional axis has also been confirmed in a variety of diseases. Guan et al.^29^ found that hsa_circ_0016788 regulates hepatocellular carcinoma tumorigenesis through miR‐486/CDK4 pathway. Xiang et al.^30^ showed that CircRNA‐CIDN mitigated compression loading‐induced damage in human nucleus pulposus cells via miR‐34a‐5p/SIRT1 axis, providing a potential therapeutic strategy for the treatment of intervertebral disc degeneration. In addition, Jiang et al.^31^ found that hsa_circ_0000658 can inhibit osteosarcoma cell proliferation and migration via the miR‐1227/IRF2 axis. However, it should be noted that the field of circRNAs is still a new area, and there is no evidence demonstrating the functions of circMARS. Zhu et al.^32^ studied the biological function and potential mechanism of circRNA through GO, KEGG pathway analysis, and circRNA‐miRNA network in the formation of OS resistance. Findings of the GO enrichment and KEGG pathway analysis in this study revealed that they are associated with the lysine degradation pathway and histone methyltransferase activity. Previous studies have also identified the role of histone modifications in ADLI progression.^2^, ^3^ And a study implicated enhancer of zest 1 (EZH1) and EZH2 are responsible for (tri‐/di‐) methylation on histone 3 lysine 27 (H3K27me2/3) in liver regeneration.^33^ According to Bae et al., combined loss of EZH1 and EZH2 in hepatocytes of mice was associated with spontaneous liver damage. Therefore, we performed cytology experiments to explore the functional axis of circMARS – miR‐3157‐5p/‐6808‐5p/‐6874‐3p – KMT2C – EGFR based on the bioinformatics analyses, existing theoretical data, and results of the dual‐luciferase reporter assay. First, the expression of circMARS in ADLI cells was increased, compared with the control group consistent with the microarray results. Second, siRNA was shown to knock down circMARS, and the downregulation was confirmed in the qRT‐PCR experiments. In the drug +si‐circMARS groups, the levels of ALT and AST were significantly higher than the drug‐only groups. Cell survival rate was also reduced, and the cell compensatory and repair regeneration ability was lost. Further, the expression of related miRNAs in the functional axis was detected. The relative expression levels of miR‐3157‐5p, miR‐6808‐5p, and miR‐6874‐3p in the ADLI cells were lower than in the control group. In addition, the miRNAs expression level in the drug +si‐circMARS groups was significantly higher than in the control group. KMT2C is a downstream target gene regulated by miR‐3157‐5p, miR‐6808‐5p, and miR‐6874‐3p. In the drug‐treated groups, a low expression of miRNAs was shown to promote the expression of KMT2C. KMT2C plays a role in histone modification^34^, ^35^ and promotes EGFR involvement in hepatocyte regeneration and repair. Liver regeneration is an important healing process in response to liver injury. Rampias et al.^36^ found that the downregulation of KMT2C in bladder cancer cells leads to extensive changes in epigenesis and the expression of DNA damage response and repair genes. MiRNAs have many target genes. In addition to KMT2C, miR‐3157‐5p, miR‐6808‐5p, and miR‐6874‐3p also target GRIN2B, which belongs to the amphetamine and nicotine addiction pathways. Previous studies have focused on the role of GRIN2B in cognition dysfunction. Thus, further studies are needed to determine if GRIN2B plays a role in hepatocyte injury. CircMARS knockdown experiment was shown to increase the expression of related miRNAs, decrease expression of the KMT2C gene and EGFR protein, aggravate cell damage, and reduce cell survival rate. Various studies have shown that the EGFR signalling pathway is involved in the occurrence and progression of liver cirrhosis and hepatocellular carcinoma and plays a crucial role in hepatocyte proliferation and liver regeneration.^37^, ^38^ It is known that circMARS has been shown to act as a miRNA sponge inhibiting the expression of miRNA, thereby exerting a positive regulatory effect on the downstream target genes. The compensatory effect of circMARS in ADLI was further demonstrated by knocking down the expression of circMARS. To further confirm the compensatory repair mechanism of the circMARS–miR‐3157‐5p/‐6808‐5p/‐6874‐3p–KMT2C–EGFR functional axis in ADLI, we detected circMARS, miRNAs, KMT2C gene, and EGFR protein levels in the self‐controlled cohort. The self‐control cohort was designed with samples from 35 patients on anti‐TB treatment before and after ADLI, as this reduces bias between individuals making the results more convincing. The qRT‐PCR study findings revealed that the expression of circMARS and KMT2C genes was increased in liver injury. However, the expressions of miR‐3157‐5p, miR‐6808‐5p, and miR‐6874‐3p were decreased. The ELISA method detected a significant increase in the serum EGFR protein levels, suggesting that the liver undergoes compensatory repair in the hepatotoxicity induced by anti‐TB drugs. There were some limitations in this study. First, the data from this study should be replicated in large‐scale studies incorporating people of different races or from different regions. Moreover, the specificity of circMARS in distinguishing ADLI from chronic hepatitis, cirrhosis, liver cancer, and other liver diseases and the economic cost of exploitation as a diagnostic biomarker should also be evaluated.^34^

In conclusion, circMARS may participate in the compensatory repair of anti‐TB drug‐induced liver injury through the circMARS–miR‐6808‐5p/‐6874‐3p/‐3157‐5p–KMT2C–EGFR function axis. CircMARS also shows promising potential as a biomarker for ADLI diagnosis in TB patients.

## CONFLICTS OF INTEREST

The authors confirm that there are no conflicts of interest.

## AUTHOR CONTRIBUTION

**Biao Li:** Conceptualization (equal); Data curation (equal); Investigation (equal); Methodology (lead); Project administration (equal); Resources (equal); Software (equal); Validation (lead); Visualization (equal); Writing – original draft (lead); Writing – review & editing (equal). **Qi Ren:** Data curation (equal); Investigation (lead); Project administration (equal); Resources (equal); Supervision (lead). **Yuhong Li:** Conceptualization (equal); Data curation (lead); Investigation (lead); Methodology (equal); Project administration (lead); Resources (equal); Supervision (equal); Validation (equal). **Shenqian Tian:** Data curation (equal); Investigation (equal); Methodology (equal); Resources (equal). **Yingzhi Chong:** Investigation (equal); Resources (equal); Software (supporting). **Shufeng Sun:** Conceptualization (equal); Investigation (equal); Project administration (equal); Resources (equal); Supervision (equal). **Fumin Feng:** Conceptualization (lead); Funding acquisition (lead); Project administration (lead); Supervision (lead); Writing – review & editing (lead).

## Supporting information

Supplementary Material
