# Supplementary material for: Screening differential circular RNA expression profiles reveals the regulatory role of circMARS in anti‐tuberculosis drug‐induced liver injury
Source: J Cell Mol Med. 2022 Jan 14;26(4):1050–9. doi: 10.1111/jcmm.17157 (PMC8831982; doi:10.1111/jcmm.17157)

**Supplementary files**

**Expression and mechanism of circMARS in ADLI**

**Table S1 Sequence of primers and siRNAs**

| Symbol name | | 5’- > -3’ |
| --- | --- | --- |
| circMARS (divergent primer) | Forward | ACCACAGCTTGAGTCGTCAG |
|  | Reverse | GGGACCACACAATCAGGGAG |
| MARS (convergent primer) | Forward | GGCCTAAGGTCCCTGTCTTG |
|  | Reverse | CCCCACAAAACAATGTCGGC |
| KMT2C | Forward | TGGGTTCACCTAGAGTGTGAC |
|  | Reverse | CTGGCTGTAAACGATCCATCTC |
| miR-3157-5p | Stem-loop | GTCGTATCCAGTGCGTGTCGTGGAGTCGGCAATTGCACTGGATACGACAGACTGC |
|  | Forward | CAACTATTCAGCCAGGCTAGT |
|  | Reverse | CAGTGCGTGTCGTGGAGT |
| miR-6808-5p | Stem-loop | GTCGTATCCAGTGCGTGTCGTGGAGTCGGCAATTGCACTGGATACGACCATGGTC |
|  | Forward | CAACTACAGGCAGGGAGGT |
|  | Reverse | CAGTGCGTGTCGTGGAGT |
| miR-6874-3p | Stem-loop | GTCGTATCCAGTGCGTGTCGTGGAGTCGGCAATTGCACTGGATACGACCTAGAGT |
|  | Forward | CAACTACAGTTCTGCTGTTCTG |
|  | Reverse | CAGTGCGTGTCGTGGAGT |
| GAPDH | Forward | GAAGGTCGGAGTCAACGGATT |
|  | Reverse | CCTGGAAGATGGTGATGGGAT |
| U6 | RT | AACGCTTCACGAATTTGCGTG |
|  | Forward | GCTCGCTTCGGCAGCACA |
|  | Reverse | GAGGTATTCGCACCAGAGGA |
| si-circMARS | Sense | CGCCUACCUCCCUGAUUGUGUTT |
|  | Antisense | ACACAAUCAGGGAGGUAGGCGTT |
| siRNA-control | Sense | GUUACCUCGUCAGUCUCUCCGTT |
|  | Antisense | CGGAGAGACUGACGAGGUAACTT |

**Table S2 Basic information of Anti-TB patients in circRNA expression profile detection**

|  | | ADLI | NON-ADLI |
| --- | --- | --- | --- |
| Gender (n) | Man | 13 | 3 |
|  | Woman | 13 | 3 |
| Age (year) | | 37.81±21.67 | 38.06±18.08 |
| BMI (kg/m^2^) | | 19.84±1.80 | 20.75±3.22 |

**Table S3 Significantly regulated circRNAs by ADLI in cells and human serum**

| circRNA ID | Regulation | Fold Change  (Serum) | *p* value | Best Transcript | Gene Symbol |
| --- | --- | --- | --- | --- | --- |
| hsa_circ_0010996 | up | 17.93 | <0.01 | NM_024887 | DHDDS |
| hsa_circ_0016762 | up | 13.36 | <0.01 | NM_053052 | SNAP47 |
| hsa_circ_0023117 | up | 12.30 | <0.01 | NM_000852 | GSTP1 |
| hsa_circ_0041957 | up | 11.87 | <0.01 | NM_001080424 | KDM6B |
| hsa_circ_0033188 | up | 8.69 | <0.01 | NM_004184 | WARS |
| hsa_circ_0089045 | up | 3.28 | <0.01 | NM_014064 | METTL11A |
| hsa_circ_0027252 | up | 2.27 | <0.01 | NM_004990 | MARS |
| hsa_circ_0004282 | down | 2.00 | <0.01 | NM_001127322 | CBX5 |
| hsa_circ_0071486 | down | 2.01 | <0.01 | NM_001080477 | ODZ3 |
| hsa_circ_0039511 | down | 2.02 | <0.01 | NM_000339 | SLC12A3 |
| hsa_circ_0066907 | down | 2.04 | <0.01 | NM_007085 | FSTL1 |
| hsa_circ_0065034 | down | 2.04 | <0.01 | NM_020242 | KIF15 |
| hsa_circ_0000630 | down | 2.05 | <0.01 | NM_033028 | BBS4 |
| hsa_circ_0047043 | down | 2.08 | <0.01 | NM_003799 | RNMT |
| hsa_circ_0058974 | down | 2.10 | <0.01 | NR_033841 | LOC200772 |
| hsa_circ_0069236 | down | 2.12 | <0.01 | NM_001145847 | PROM1 |
| hsa_circ_0090153 | down | 2.16 | <0.01 | NM_016937 | POLA1 |
| hsa_circ_0080194 | down | 2.21 | <0.01 | NM_138295 | PKD1L1 |
| hsa_circ_0091576 | down | 2.22 | <0.01 | NM_001170704 | MBNL3 |
| hsa_circ_0080101 | down | 2.22 | <0.01 | NM_033054 | MYO1G |
| hsa_circ_0053447 | down | 2.25 | <0.01 | NM_016252 | BIRC6 |
| hsa_circ_0080707 | down | 2.31 | <0.01 | NM_005338 | HIP1 |
| hsa_circ_0034180 | down | 2.32 | <0.01 | NM_024490 | ATP10A |
| hsa_circ_0047730 | down | 2.38 | <0.01 | NM_001083962 | TCF4 |
| hsa_circ_0027155 | down | 2.38 | <0.01 | NM_002332 | LRP1 |
| hsa_circ_0037000 | down | 2.39 | <0.01 | NM_001271 | CHD2 |
| hsa_circ_0081550 | down | 2.41 | <0.01 | NM_015908 | SRRT |
| hsa_circ_0082427 | down | 2.41 | <0.01 | NM_020911 | PLXNA4 |
| hsa_circ_0012791 | down | 2.42 | <0.01 | NM_181712 | KANK4 |
| hsa_circ_0023979 | down | 2.47 | <0.01 | NM_001814 | CTSC |
| hsa_circ_0087124 | down | 2.50 | <0.01 | TCONS_l2_00029973 | TCONS_l2_00029973 |
| hsa_circ_0073779 | down | 2.50 | <0.01 | NM_001999 | FBN2 |
| hsa_circ_0032019 | down | 2.51 | <0.01 | NM_007086 | WDHD1 |
| hsa_circ_0049283 | down | 2.57 | <0.01 | NM_001145056 | SLC44A2 |
| hsa_circ_0073795 | down | 2.59 | <0.01 | NM_001999 | FBN2 |
| hsa_circ_0081165 | down | 2.59 | <0.01 | NM_000089 | COL1A2 |
| hsa_circ_0015901 | down | 2.63 | <0.01 | NM_020443 | NAV1 |
| hsa_circ_0045826 | down | 2.65 | <0.01 | NM_018414 | ST6GALNAC1 |
| hsa_circ_0062272 | down | 2.66 | <0.01 | NR_037611 | SEPT5-GP1BB |
| hsa_circ_0029178 | down | 2.68 | <0.01 | NM_022782 | MPHOSPH9 |
| hsa_circ_0038396 | down | 2.74 | <0.01 | NM_001199053 | LOC81691 |
| hsa_circ_0058890 | down | 2.75 | <0.01 | NM_001040445 | ASB1 |
| hsa_circ_0077941 | down | 2.77 | <0.01 | NM_018945 | PDE7B |
| hsa_circ_0026173 | down | 2.79 | <0.01 | NM_175736 | FMNL3 |
| hsa_circ_0036978 | down | 2.81 | <0.01 | NM_001271 | CHD2 |
| hsa_circ_0052581 | down | 2.83 | <0.01 | NM_001165931 | RRM2 |
| hsa_circ_0062463 | down | 2.87 | <0.01 | None | None |
| hsa_circ_0019035 | down | 2.89 | <0.01 | NM_005271 | GLUD1 |
| hsa_circ_0055625 | down | 2.95 | <0.01 | NM_004418 | DUSP2 |
| hsa_circ_0052317 | down | 2.98 | <0.01 | NM_152475 | ZNF417 |
| hsa_circ_0072737 | down | 3.05 | <0.01 | NM_139168 | SREK1 |
| hsa_circ_0046925 | down | 3.09 | <0.01 | NM_022068 | PIEZO2 |
| hsa_circ_0065239 | down | 3.12 | <0.01 | NM_001206942 | CSPG5 |
| hsa_circ_0023831 | down | 3.16 | <0.01 | NM_001098816 | ODZ4 |
| hsa_circ_0064070 | down | 3.16 | <0.01 | NM_001168272 | ITPR1 |
| hsa_circ_0078800 | down | 3.17 | <0.01 | NM_052923 | SCAND3 |
| hsa_circ_0037006 | down | 3.17 | <0.01 | NM_001271 | CHD2 |
| hsa_circ_0050293 | down | 3.19 | <0.01 | TCONS_l2_00013305 | TCONS_l2_00013305 |
| hsa_circ_0059125 | down | 3.24 | <0.01 | NM_152783 | D2HGDH |
| hsa_circ_0010907 | down | 3.26 | <0.01 | NM_001199014 | C1orf201 |
| hsa_circ_0071397 | down | 3.29 | <0.01 | NM_001012967 | DDX60L |
| hsa_circ_0066444 | down | 3.32 | <0.01 | NM_182920 | ADAMTS9 |
| hsa_circ_0050664 | down | 3.38 | <0.01 | NM_021232 | PRODH2 |
| hsa_circ_0062573 | down | 3.39 | <0.01 | NM_000853 | GSTT1 |
| hsa_circ_0035957 | down | 3.44 | <0.01 | NM_001144823 | DENND4A |
| hsa_circ_0083576 | down | 3.49 | <0.01 | NM_022749 | FAM160B2 |
| hsa_circ_0061141 | down | 3.54 | <0.01 | NM_022082 | SLC17A9 |
| hsa_circ_0042113 | down | 3.66 | <0.01 | NM_001303 | COX10 |
| hsa_circ_0057135 | down | 3.72 | <0.01 | NM_001077269 | WIPF1 |
| hsa_circ_0012054 | down | 3.72 | <0.01 | NM_015284 | SZT2 |
| hsa_circ_0082285 | down | 3.73 | <0.01 | NM_005631 | SMO |
| hsa_circ_0047202 | down | 3.77 | <0.01 | NM_198129 | LAMA3 |
| hsa_circ_0053291 | down | 3.80 | <0.01 | NM_032434 | ZNF512 |
| hsa_circ_0018453 | down | 3.81 | <0.01 | NM_032199 | ARID5B |
| hsa_circ_0012243 | down | 3.84 | <0.01 | NM_002482 | NASP |
| hsa_circ_0006765 | down | 3.90 | <0.01 | NM_024692 | CLIP4 |
| hsa_circ_0050273 | down | 3.92 | <0.01 | NM_031218 | ZNF93 |
| hsa_circ_0044407 | down | 3.98 | <0.01 | NM_016428 | ABI3 |
| hsa_circ_0028698 | down | 4.10 | <0.01 | NM_001206999 | CIT |
| hsa_circ_0037387 | down | 4.14 | <0.01 | NR_003142 | SNHG9 |
| hsa_circ_0012743 | down | 4.15 | <0.01 | NM_001085487 | MYSM1 |
| hsa_circ_0008591 | down | 4.29 | <0.01 | NM_053025 | MYLK |
| hsa_circ_0037199 | down | 4.45 | <0.01 | NM_005632 | SOLH |
| hsa_circ_0036920 | down | 4.60 | <0.01 | NM_003981 | PRC1 |
| hsa_circ_0068266 | down | 4.66 | <0.01 | NM_003907 | EIF2B5 |
| hsa_circ_0034332 | down | 4.84 | <0.01 | NM_014783 | ARHGAP11A |
| hsa_circ_0030071 | down | 4.85 | <0.01 | NM_024561 | NAA16 |
| hsa_circ_0092321 | down | 4.85 | <0.01 | NM_133374 | ZNF618 |
| hsa_circ_0079739 | down | 4.86 | <0.01 | NR_037598 | INMT-FAM188B |
| hsa_circ_0031953 | down | 4.93 | <0.01 | NM_001130701 | STYX |
| hsa_circ_0092087 | down | 4.97 | <0.01 | NM_017514 | PLXNA3 |
| hsa_circ_0074181 | down | 5.04 | <0.01 | NR_036536 | SNHG4 |
| hsa_circ_0025088 | down | 5.04 | <0.01 | NM_000552 | VWF |
| hsa_circ_0056131 | down | 5.06 | <0.01 | NM_153214 | FBLN7 |
| hsa_circ_0092295 | down | 5.07 | <0.01 | NM_012398 | PIP5K1C |
| hsa_circ_0091643 | down | 5.09 | <0.01 | NM_001171876 | MCF2 |
| hsa_circ_0081101 | down | 5.15 | <0.01 | NM_000089 | COL1A2 |
| hsa_circ_0018320 | down | 5.24 | <0.01 | NM_020945 | WDFY4 |
| hsa_circ_0031816 | down | 5.49 | <0.01 | NM_001663 | ARF6 |
| hsa_circ_0058888 | down | 5.64 | <0.01 | NM_001040445 | ASB1 |
| hsa_circ_0087818 | down | 5.95 | <0.01 | NM_005502 | ABCA1 |
| hsa_circ_0012523 | down | 6.00 | <0.01 | NM_004153 | ORC1 |
| hsa_circ_0058484 | down | 6.41 | <0.01 | NM_014689 | DOCK10 |
| hsa_circ_0089633 | down | 7.09 | <0.01 | NM_016219 | MAN1B1 |
| hsa_circ_0040959 | down | 7.30 | <0.01 | NM_014427 | CPNE7 |
| hsa_circ_0038874 | down | 7.72 | <0.01 | NM_004320 | ATP2A1 |
| hsa_circ_0061162 | down | 7.82 | <0.01 | NM_001037335 | PRIC285 |
| hsa_circ_0064044 | down | 7.87 | <0.01 | NM_001168272 | ITPR1 |
| hsa_circ_0071288 | down | 8.52 | <0.01 | NM_001130067 | TRIM2 |
| hsa_circ_0056231 | down | 9.41 | <0.01 | None | None |
| hsa_circ_0021272 | down | 9.71 | <0.01 | NM_198516 | GALNTL4 |
| hsa_circ_0000301 | down | 10.41 | <0.01 | NM_001080547 | SPI1 |
| hsa_circ_0021214 | down | 11.30 | <0.01 | NM_030962 | SBF2 |

**Table S4 Target miRNAs of circMARS**

| 98 target miRNAs of circMARS | | | | |
| --- | --- | --- | --- | --- |
| hsa-miR-3157-5p | hsa-miR-6808-5p | hsa-miR-6874-3p | hsa-miR-4743-3p | hsa-let-7e-5p |
| hsa-let-7a-5p | hsa-let-7d-5p | hsa-let-7i-5p | hsa-miR-106b-3p | hsa-miR-1184 |
| hsa-miR-1236-3p | hsa-miR-125b-1-3p | hsa-miR-1292-5p | hsa-miR-1294 | hsa-miR-1301-3p |
| hsa-miR-1303 | hsa-miR-1321 | hsa-miR-135a-3p | hsa-miR-150-3p | hsa-miR-196b-5p |
| hsa-miR-214-3p | hsa-miR-2355-5p | hsa-miR-3064-5p | hsa-miR-3127-3p | hsa-miR-3127-5p |
| hsa-miR-3154 | hsa-miR-3158-3p | hsa-miR-3619-5p | hsa-miR-3649 | hsa-miR-365a-5p |
| hsa-miR-365b-5p | hsa-miR-3675-5p | hsa-miR-3918 | hsa-miR-3937 | hsa-miR-4270 |
| hsa-miR-431-3p | hsa-miR-4320 | hsa-miR-4436b-3p | hsa-miR-4458 | hsa-miR-4487 |
| hsa-miR-4505 | hsa-miR-4632-5p | hsa-miR-4640-3p | hsa-miR-4654 | hsa-miR-4660 |
| hsa-miR-4685-3p | hsa-miR-4685-5p | hsa-miR-4690-5p | hsa-miR-4691-3p | hsa-miR-4709-3p |
| hsa-miR-4715-5p | hsa-miR-4724-5p | hsa-miR-4725-3p | hsa-miR-4739 | hsa-miR-4756-5p |
| hsa-miR-4763-3p | hsa-miR-4776-5p | hsa-miR-4793-5p | hsa-miR-5006-5p | hsa-miR-5088-3p |
| hsa-miR-5088-5p | hsa-miR-6069 | hsa-miR-6089 | hsa-miR-6127 | hsa-miR-6133 |
| hsa-miR-647 | hsa-miR-6504-5p | hsa-miR-6512-3p | hsa-miR-6516-5p | hsa-miR-664b-3p |
| hsa-miR-6720-5p | hsa-miR-6735-5p | hsa-miR-6754-5p | hsa-miR-6774-5p | hsa-miR-6775-5p |
| hsa-miR-6777-5p | hsa-miR-6779-3p | hsa-miR-6779-5p | hsa-miR-6781-5p | hsa-miR-6798-5p |
| hsa-miR-6799-5p | hsa-miR-6802-5p | hsa-miR-6825-5p | hsa-miR-6837-5p | hsa-miR-6861-5p |
| hsa-miR-6876-5p | hsa-miR-6879-5p | hsa-miR-6880-5p | hsa-miR-7106-5p | hsa-miR-7113-5p |
| hsa-miR-7162-5p | hsa-miR-765 | hsa-miR-7843-5p | hsa-miR-7847-3p | hsa-miR-8081 |
| hsa-miR-92b-5p | hsa-miR-937-5p | hsa-miR-942-5p |  |  |

**Table S5 Basic information of 35 ADLI patients in self-control subject**

|  | | TB patients |
| --- | --- | --- |
| Gender (n) | Man | 26 |
|  | Woman | 9 |
| Age (year) | | 39.80±16.54 |
| BMI (kg/m^2^) | | 17.40±1.21 |
| the time interval between  before and during ADLI (day) | | 21.09±15.68 |
| Drinking (n) | Yes | 31 |
|  | No | 4 |
| Smoking (n) | Yes | 5 |
|  | No | 30 |

**Fig. S1 The characteristic of circMARS**

(**a**)The sequencing map of circMARS PCR products. (**b**)Divergent primers amplify circMARS in cDNA but not gDNA. M: markes; R+/-: with or without RNase R; gDNA: genomic DNA.


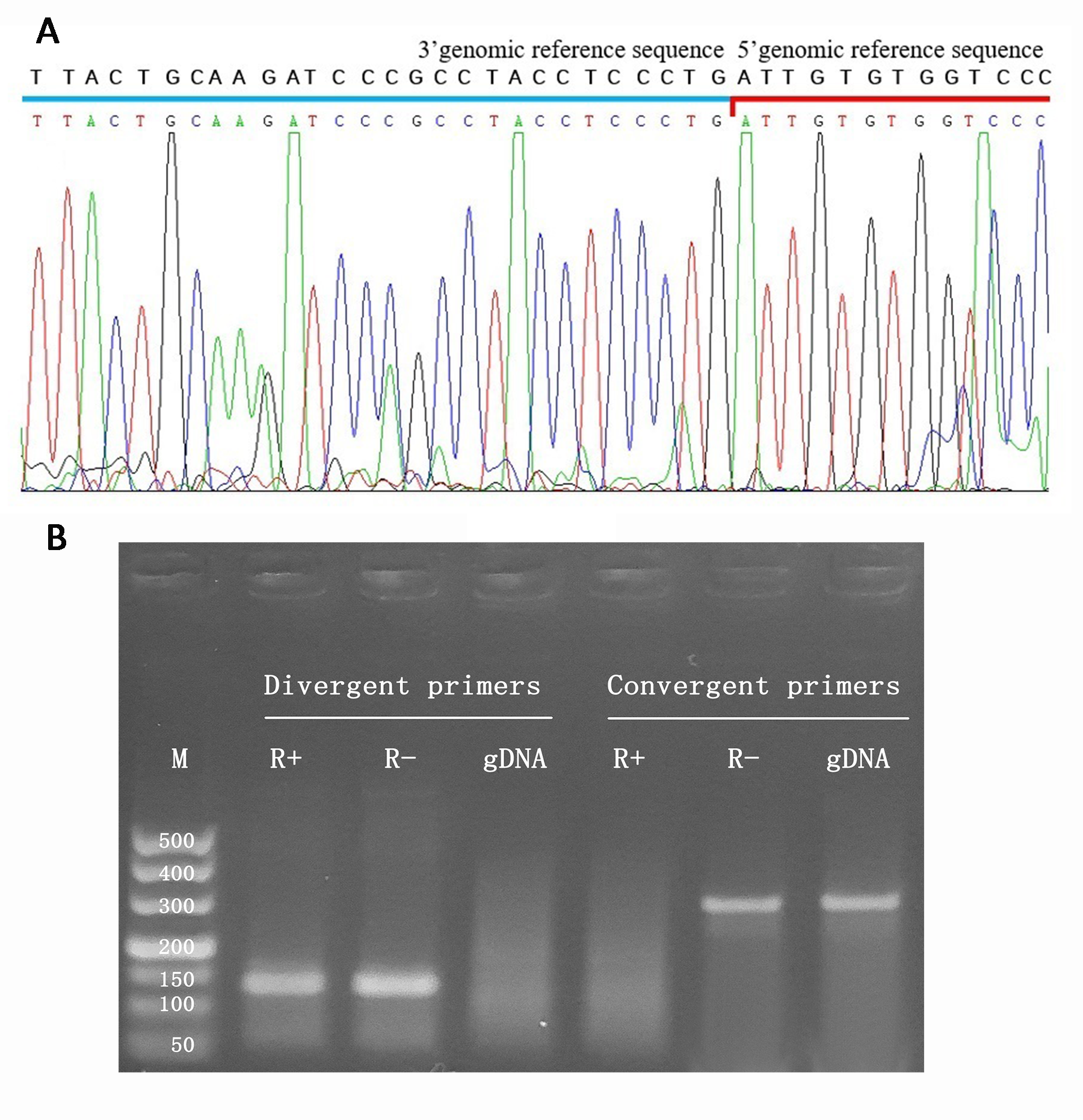


**Fig. S2 KMT2C in the Lysine degradation pathway (hsa00310)**


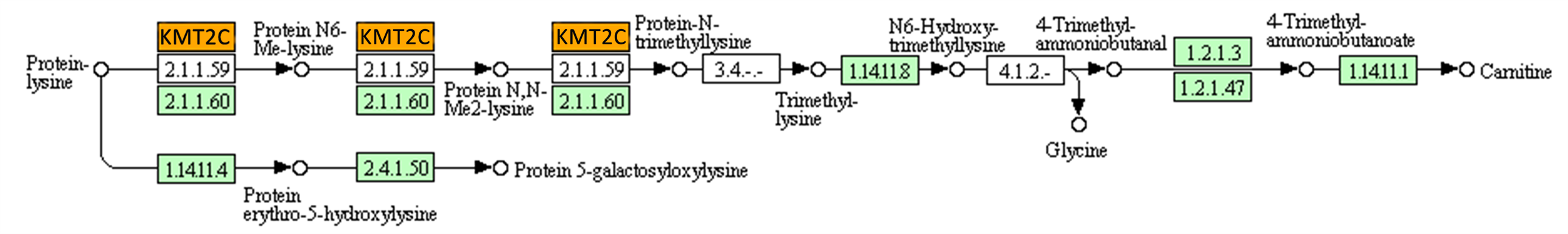


**Fig. S3 Gene ontology of histone methyltransferase activity (GO: 0042800)**


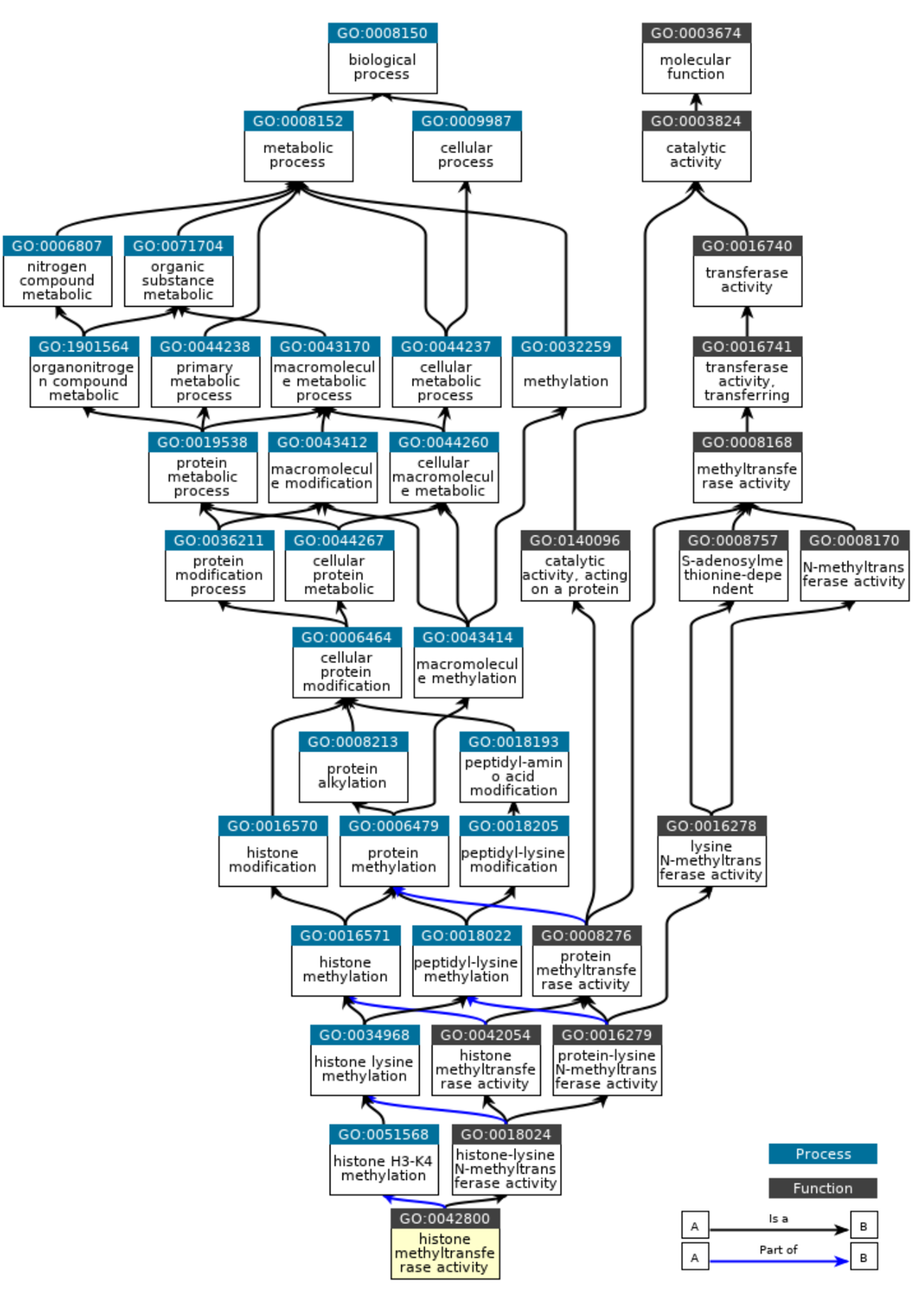


**Fig. S4 The luciferase activity of wild or mutant type circMARS after transfection with miR-3157-5p/-6808-5p/-6874-3p mimics or miR-NC**

**cell lines**


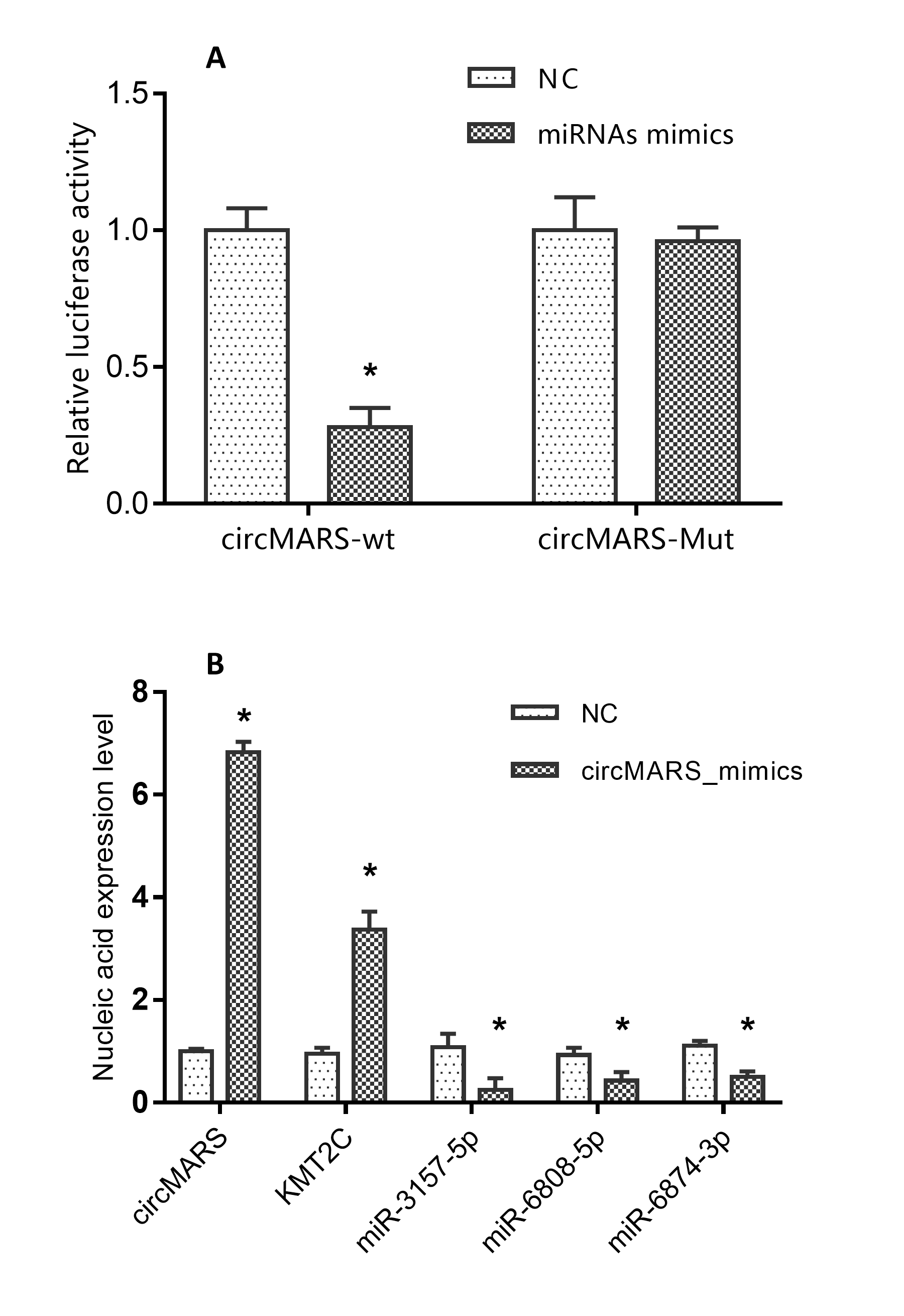

Supplement: Supplementary file 1 — Supplementary Material [file JCMM-26-1050-s001.docx]
